# Video intervention increases participation of black breast cancer patients in therapeutic trials

**DOI:** 10.1038/s41523-017-0039-1

**Published:** 2017-09-18

**Authors:** Brandi N. Robinson, Antoinette F. Newman, Eshetu Tefera, Pia Herbolsheimer, Raquel Nunes, Christopher Gallagher, Pamela Randolph-Jackson, Adedamola Omogbehin, Asma Dilawari, Paula R. Pohlmann, Mahsa Mohebtash, Young Lee, Yvonne Ottaviano, Avani Mohapatra, Filipa Lynce, Richard Brown, Mihriye Mete, Sandra M. Swain

**Affiliations:** 1 MedStar Health Research Institute, Hyattsville, MD USA; 2 Washington Cancer Institute, MedStar Washington Hospital Center, Washington, DC USA; 3 Lombardi Comprehensive Cancer Center, MedStar Georgetown University Hospital, Washington, DC USA; 4 MedStar Union Memorial Hospital, Baltimore, MD USA; 5 MedStar Harbor Hospital, Baltimore, MD USA; 6 MedStar Franklin Square Medical Center, Baltimore, MD USA; 7 Virginia Commonwealth University School of Medicine, Richmond, VA USA

## Abstract

There is a striking racial and ethnic disparity in incidence and mortality of cancer yet minorities remain markedly underrepresented in clinical trials. This pilot study set out to determine the impact of a 15-min culturally tailored educational video on three outcomes relating to clinical trials: likely participation, attitudes (assessed based on six barriers), and actual enrollment. Breast cancer patients with Stage I-III, if diagnosed within previous 6 months, or metastatic disease who self-identified as black or African American were invited to participate. The primary outcome measure was the decision to participate in a therapeutic clinical trial after the intervention. Patients’ intention to enroll on a therapeutic clinical trial and the change in attitudes toward clinical trials were measured by the previously developed Attitudes and Intention to Enroll in Therapeutic Clinical Trials (AIET) questionnaire. Of the 200 patients that participated, 39 (19.5%) patients signed consent to participate in a therapeutic clinical trial; 27 (13.5%) patients enrolled, resulting in a 7.5% increase from our baseline comparison of 6% clinical trial enrollment rate in black cancer patients (*p* < .001). Pre-test versus post-test assessment demonstrated the proportion of patients expressing likelihood to enroll in a therapeutic trial following the intervention increased by 14% (*p* < .001). Among 31 AIET items, 25 (81%) showed statistically significant and positive change post-intervention. The findings suggest the promising utility of a culturally tailored video intervention for improving black patients’ attitudes regarding clinical trial participation and resultant enrollment. Future efforts should continue to target facilitators of population-specific recruitment, enrollment, and retention in therapeutic and non-therapeutic clinical trials.

## Educational video boosts trial enrollment among black patients with breast cancer

A culturally tailored educational video can boost participation among black patients in clinical trials of new breast cancer treatments. A US team led by Sandra Swain from Georgetown University Medical Center in Washington, DC, created a 15-min video designed to address six of the concerns commonly cited by blacks about human subjects research. The researchers showed the video to 200 black patients, and saw a large bump in the number of women willing to sign up for a therapeutic trial. On average, only 6% of black cancer patients typically enroll in clinical trials. But in the video intervention study, 19.5% agreed to participate and then 13.5% went ahead with a trial. Video watchers also reported a positive change in their attitude toward clinical research. The study points to the need for population-specific recruitment efforts.

## Introduction

Tremendous strides have been made in the prevention and treatment of cancer from clinical research since enactment of the NIH Revitalization Act.^1–3^ Much of this success can be attributed to the willingness of cancer patients to join clinical trials (CTs). Yet, the accrual rates of all cancer patients to CTs remain low, with estimates reaching as low as 5% of eligible patients joining trials and many studies indicating even lower rates of participation among minority populations, including black cancer patients.^4–7^ Low rates of accrual of minority populations to cancer CTs slows the progress of promising new treatments, represents a threat to the generalizability of newly developed cancer treatments, and suggests inequity in access to the latest treatments, which represents a significant healthcare disparity.^8–13^

Barriers to minority patients’ participation in CTs include systemic or system-level factors,^7, 14–23^ such as strict exclusion criteria,^7, 14, 16, 18, 20, 21, 23^ cost,^7, 14–18, 20, 22, 23^ transportation,^7, 16, 18, 22, 23^ and convenience,^7, 15–18, 22^ which limit opportunities to participate.^10^ Racial differences in patient barriers to participation are also due in part to both clinical (e.g., trial side effects ^7, 15–17^) and non-clinical factors such as those relating to lack of awareness/knowledge of CTs available,^7, 15, 16, 18, 20, 21, 24–26^ mistrust,^16–20, 22, 23^ family pressures,^7, 15, 16, 18, 23^ religious beliefs,^15, 19, 22, 23^ and poor patient-physician communication.^16, 18, 20, 23^ Additionally, provider-related barriers can represent significant hurdles, specifically provider perspective regarding socio-demographic and medical factors relating to education, compliance and eligibility,^16, 18, 20, 21, 24–27^ as well as the particularly difficult task of talking with ill and vulnerable cancer patients about CTs.^20, 28, 29^ Further, a paucity of culturally relevant information is available to minority patients that is evident in the lack of trial knowledge and understanding of important trial procedures, such as dose escalation and randomization.^16–19, 22, 27, 30, 31^

To address modifiable issues of low accrual of black cancer patients to CTs, a 15-minute culturally targeted video designed to impact six specific attitudes of black cancer patients toward therapeutic CTs was developed. Our results in a prior study of 108 patients with any cancer revealed that this simple video intervention positively impacted patients’ intentions to enroll in a CT (36% had a positive change) and demonstrated improvements in all six attitudinal barriers measured.^32^

This study, INSPIRE-BrC (INcreaSing Participation In Research-Breast Cancer), set out to assess whether the video intervention could impact actual clinical trial enrollment (if eligible and available). The study is guided by the Theory of Planned Behavior that focuses on constructs concerned with individual motivational factors as determinants of the likelihood of performing a specific behavior, which in this case, is enrolling in a therapeutic CT.^33^ The primary aim of the current study was to explore the ability of the short video to impact black breast cancer patients’ actual participation in a therapeutic CT. Our secondary aims were to explore the capacity of the video to influence black breast cancer patients’ (a) intentions to participate in a CT; and, (b) attitudes towards CTs. Changes in (a) and (b) were measured using the The Attitudes and Intention to Enroll in Therapeutic Clinical Trials (AIET) questionnaire (Supplementary Table 1) administered at three separate time points: pre-video, immediately following the video (post-test), and between 7 and 21 days post-video intervention (follow-up).

## Results

**Fig. 1:**
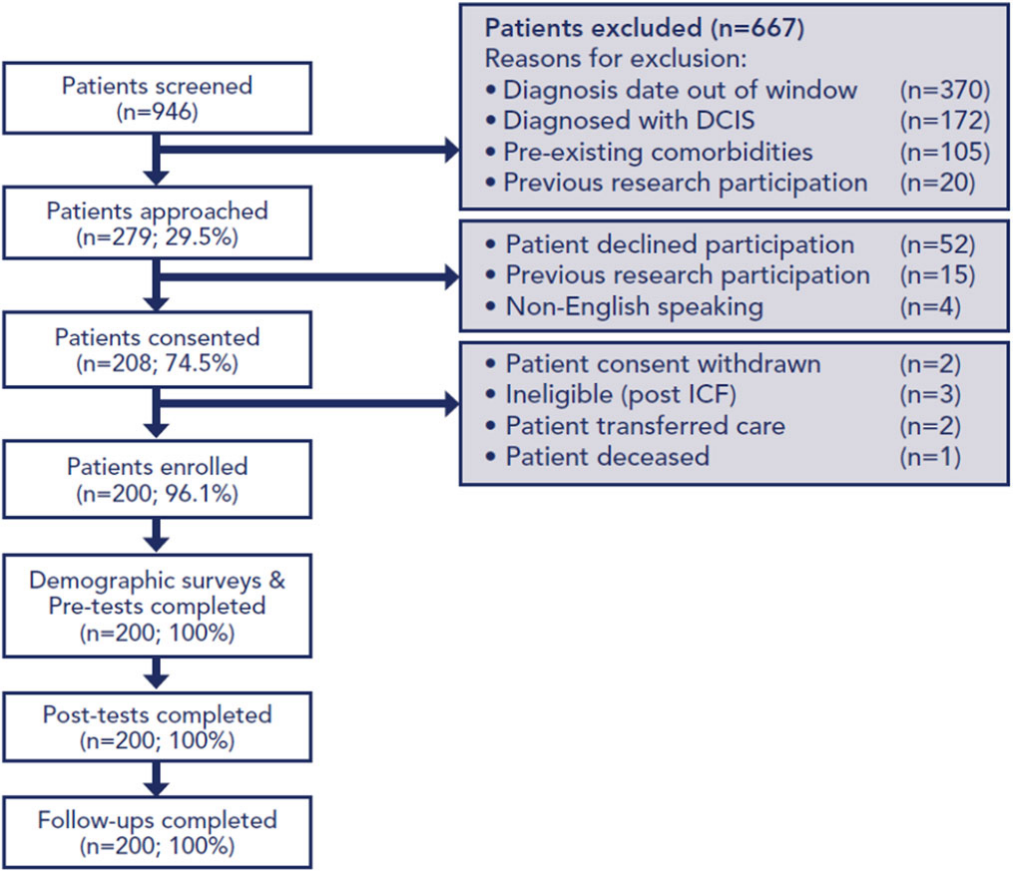
Flowchart diagram of participation process. Flowchart diagram outlining patients included in the analysis, reasons for patient exclusion, and participation process. DCIS ductal carcinoma in situ, ICF informed consent form

From March 2014 to September 2015, 946 patients were screened (Fig. 1). A total of 279 patients were approached to participate; however, of these, four were non-English speaking (1.4%), 15 (5.3%) had participated in previous research, and 52 (18.6%) declined participation stating they were overwhelmed by their diagnosis/needed to focus on their care or were not interested due to time. Of the 208 patients who signed consent, two withdrew consent; three were found to be ineligible after signing the consent (two had previously participated in a therapeutic trial and one was determined to have been diagnosed more than 6 months prior); two transferred to non-MedStar Health hospitals; and one died before participating in the intervention.

Baseline sociodemographic and clinical characteristics of the patients that completed the intervention and all three AIET assessments (*n* = 200) are listed in Table 1. Mean age of the participants was 59 years, 74.5% had stage I-III disease, 29% were married, 83.5% had one or more children, and 29% attended some college or technical school.

**Table 1:** Sociodemographic and clinical characteristics

Characteristic	No. (%) *N* = 200
Age, mean ± SD, years	59.1 ± 12.2
Race	
African American	128 (64)
Black	61 (30.5)
African ancestry	1 (0.5)
Caribbean or West Indian ancestry	8 (4)
Other^a^	2 (1)
Were you born in the United States?	
Yes	188 (94)
Marital status	
Never married	54 (27)
Married	58 (29)
Marriage equivalent	1 (0.5)
Widowed	31 (15.5)
Separated/divorced	53 (26.5)
Declined to answer	3 (1.5)
Children	
None	33 (16.5)
1 or more	167 (83.5)
Education	
Less than high school	27 (13.5)
High school graduate	43 (21.5)
Some college or technical school	58 (29)
College graduate	45 (22.5)
Post graduate	27 (13.5)
Religion	
Baptist/Freewill Baptist	99 (49.5)
Catholic	26 (13)
Methodist	13 (6.5)
Pentecostal/holiness	16 (8)
Other^b^	44 (22)
Declined to answer	2 (1)
Income	
<$30,000	76 (38)
$30,000–$59,999	44 (22)
$60,000–$99,999	35 (17.5)
>$100,000	26 (13)
Declined to answer	19 (9.5)
Family history of cancer	
Yes	146 (74)
Stage of cancer	
I, II, III	149 (74.5)
IV	51 (25.5)

*SD* standard deviation

^a^ Two patients self-identified as black but both were biracial (part Native American)

^b^ Includes Episcopalian, Jehovah’s Witness, Presbyterian, Muslim, as well as others not listed on demographic survey or no religious affiliation

### Primary outcome

*Results for Aim 1*: Of the 200 patients who participated in the study, 39/200 (19.5%) signed consent and/or enrolled onto a therapeutic trial (Table 2). Patients were included who signed consent regardless if they enrolled in a trial, as the initial signing demonstrated agreement to participate in a therapeutic CT. A total of 27/200 (13.5%) were enrolled in a study within 6 months of viewing the video, yielding a highly significant increase from baseline (*p* < .001). Eleven of the patients who signed consent but did not enroll were found to be ineligible after signing based on new laboratory findings and/or a change in disease status. A single patient withdrew her consent because she did not want to participate in a required component of the study procedures.

**Table 2:** Primary outcome measures

Patients eligible	No. (%) (*N* = 200)
Signed consent/enrolled in CT	39 (19.5)
Enrolled within 6 months of viewing video	27 (13.5)
versus 2012 baseline (6%)	*p* < .001
Trials available	No. (%) (*N* = 54)
Signed consent/enrolled in CT	39 (72.2)
Declined^a^	15 (27.8)

*CT* clinical trial

^a^ Declined for the following reasons: time constraints (*n* = 8); overwhelmed with diagnosis (*n* = 5); family members’ disapproval of the trial (*n* = 1); skepticism regarding intentions due to race (*n* = 1)

Therapeutic trials were available for 54/200 (27%) patients; all were approached to participate (Table 2). Of these patients, 39/54 (72.2%) who were eligible for a therapeutic trial within 6 months of the intervention signed consent/enrolled; 15 declined. A comparison of demographics among patients eligible for a trial showed advanced stage of disease as the only significant factor to the signing of consent/enrolling (*p* = .038).

### Secondary outcomes

*Results for Aim 2 (a)*: The proportion of participants expressing likelihood to enroll in a therapeutic trial significantly increased after watching the video, both immediately and 7–21 days after the intervention: 52% pre-video versus 66% post-video (*p* < .001) and 64% follow-up (*p* = .003).

*Results for Aim 2 (b)*: A significant pre-to-post-to-follow-up difference (*p* < .05) consisting of a change to more positive attitudes toward CT participation over time was demonstrated in 25 (81%) of the 31 attitudinal barrier measures (including likely trial participation) (Table 3). The measures that did not show significant change over time were: the importance of the reputation of the treatment center where a hypothetical CT is being done; trust in healthcare workers; changing one’s mind about participating in a trial at any time; researchers would only do what is stated in the consent form; people can access medical records without approval; and, there are always serious side effects to related to CTs. For these measures, patients already held more positive attitudes at baseline, which they maintained over the next two assessments.

**Table 3:** Mean responses of attitudes and intention to enroll in therapeutic clinical trials questionnaire

Question	Pre-test	Post-test	Follow-up	*p*-value
Q1. Trust in the doctor who offers you the trial	**4.52**	**4.56**	**4.52**	**0.0080**
Q2. Reputation of the treatment center where the trial is done	4.57	4.62	4.60	0.7483
Q3. I cannot trust health care workers	1.99	1.78	1.88	0.0767
Q4. I am suspicious of CTs	**2.75**	**2.17**	**2.34**	<**0.0001**
Q5. I am suspicious of information I receive from researchers	**2.59**	**2.10**	**2.16**	<**0.0001**
Q6. Most clinical research is ethical	**2.51**	**2.05**	**2.15**	<**0.0001**
Q7. Researchers do not care about me/my well being	**2.05**	**1.69**	**1.82**	**0.0002**
Q8. My doctor would not ask me to participate in a CT if he or she thought it would hurt me	**1.75**	**1.40**	**1.57**	**0.0004**
Q9. People who approve CTs make sure all participants are treated fairly	**1.96**	**1.56**	**1.60**	<**0.0001**
Q10. Might be used as a guinea pig if you were in a CT	**2.64**	**2.07**	**1.99**	<**0.0001**
Q11. I could still ask my doctors any questions that I want	**1.34**	**1.14**	**1.16**	**0.0126**
Q12. If doctors took my blood they could do tests on it they have not told me about	**2.50**	**2.09**	**2.08**	<**.0001**
Q13. I would only be agreeing to do what is explained to me in the consent form	**1.51**	**1.41**	**1.28**	**0.0041**
Q14. I could still change my mind about participating at any time	1.30	1.21	1.19	0.2522
Q15. The researchers would only do what is stated in the consent form	1.65	1.53	1.58	0.2114
Q16. Black people in CTs receive the same care from doctors and health care workers as people of other races or ethnicities on CTs	**2.28**	**1.78**	**1.83**	<**0.0001**
Q17. If I were to enroll in a CT my doctors would treat me with dignity and respect	**1.60**	**1.33**	**1.28**	<**0.0001**
Q18. Poor people are used more in research without their permission	**2.60**	**2.11**	**2.27**	<**0.0001**
Q19. How often do you think doctors prescribe medication as a way of experimenting on black patients without their knowledge or permission?	**2.24**	**1.94**	**1.95**	<**0.0001**
Q20. Black people are used more in research without their knowledge or permission	**2.52**	**2.00**	**2.03**	<**0.0001**
Q21. People can access my medical records without my approval	2.05	1.93	1.91	0.2392
Q22. My medical records are kept private	**1.87**	**1.59**	**1.67**	**0.0002**
Q23. My privacy is a major concern for the researchers	**1.83**	**1.52**	**1.49**	<**0.0001**
Q24. Personal information like my name, address and phone number will remain confidential	**1.67**	**1.47**	**1.52**	**0.0125**
Q25. Any center doing CTs has set rules to make sure my records are kept safe	**1.69**	**1.46**	**1.47**	**0.0010**
Q26. There are always serious side effects related to CTs	2.84	2.71	2.68	0.1625
Q27. If my doctor wanted me to participate in a CT, he or she would fully explain to me everything that is involved	**1.47**	**1.27**	**1.28**	**0.0006**
Q28. I can talk to my doctors to find out about participating in CTs	**1.42**	**1.34**	**1.20**	<**0.0001**
Q29. There may be benefits for me if I participate in a CT	**1.98**	**1.63**	**1.68**	<**0.0001**
Q30. There may be benefits for other people like me if I participate in a CT	**1.64**	**1.42**	**1.50**	**0.0060**
Q31. At this moment, is it likely that you would sign up to participate in a therapeutic CT?	**1.48**	**1.34**	**1.37**	<**0.0001**

For Q1 and Q2, a score of 1 = not at all important and 5 = holds very much importance; for Q3-5, Q7, Q12, Q18, Q20, Q21 and Q26, a score of 1 = strongly disagree and 5 = strongly agree; for Q10, a score of 1 = not at all likely and 5 = very likely; for Q19, a score of 1 = Never and 5 = Very often; for Q31, a score of 1 = yes and 2 = no; for all other Q#s, a score of 1 = strongly agree and 5 = strong disagree; Q#s in bold typeface are statistically significant

*AIET* attitudes and intention to enroll in therapeutic clinical trials, *CT* clinical trial, *Q#* question number on AIET

**Fig. 2:**
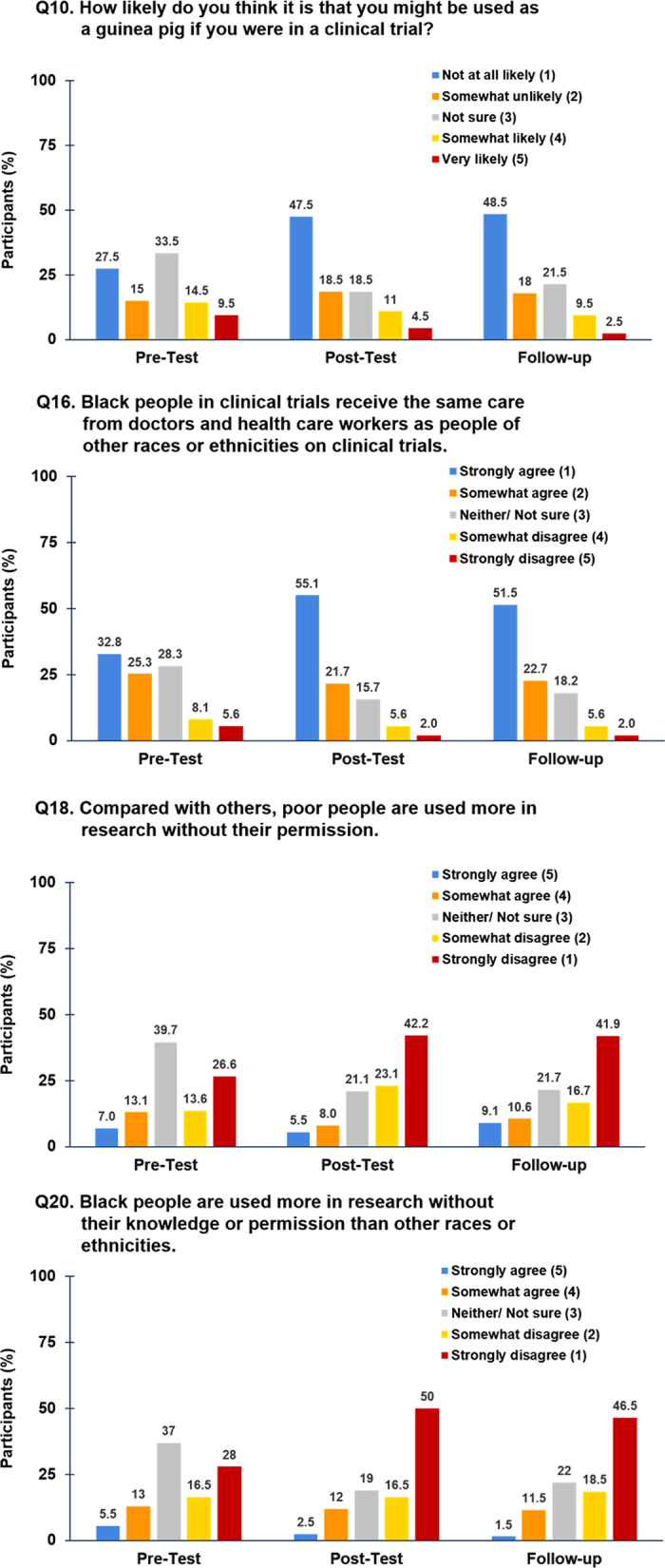
Attitudes and Intention to Enroll in Therapeutic Clinical Trials Questionnaire: Select Responses Over Time. Selected attitudinal barriers with individual response percentages across time

Prior to the video intervention, AIET data identified a number of baseline concerns including, but not limited to, 115 (57.5%) participants noting that they were Not Sure, Somewhat Likely, and Very Likely to be used as a guinea pig if they were on a CT (Fig. 2). After viewing the video, only 68 (34%) patients carried those attitudes; the shift in attitude was maintained at follow-up (*n* = 67, 33.5%). Patients also worried that investigators would treat poor and/or minority patients unfairly. When asked, *compared to others, poor people are used more in research without their permission*, on a scale of strongly agree to strongly disagree, 39.7% (*n* = 79) responded initially with Neither/Not sure and 40.2% (*n* = 80) stated they somewhat or strongly disagreed. Immediately after the intervention, 21.1% said neither/not sure and 65.3% somewhat or strongly disagreed; this positive shift in attitudes was maintained at follow-up (21.7 and 58.6%, respectively). When presented with a similar scenario, *Black people are used more in research without their knowledge or permission than other races and ethnicities*, 37% (*n* = 74) of participants had a neutral opinion by responding with Neither/Not sure at baseline and 44.5% (*n* = 89) somewhat or strongly disagreed with the statement. However, at post-test, only 19% of patients had a neutral opinion and an increasing number of patients (66.5%) noted they somewhat or strongly disagreed; this improvement in attitude was maintained at follow-up (22 and 65%, respectively).

## Discussion

Of the 200 patients that participated from the five hospital sites, approximately 20% signed consent to participate in a therapeutic trial and 14% enrolled, resulting in a statistically significant 7.5% increase from our 2012 baseline. Furthermore, 25 of 31 attitudinal barrier measures showed a significant change (*p* < .05) to more positive attitudes toward trial participation post-intervention.

Building on our previous experience, this study was guided by health behavior theories.^33^ These theories assert that individual behavior is driven by behavioral intentions, which are a function of an individual’s attitude toward the behavior; subjective norms surrounding the performance of the behavior; and, the individual’s perception of the ease with which the behavior can be performed (perceived control). We developed a tool to address attitudinal barriers that decrease black cancer patients’ participation in therapeutic CTs.^32^ This culturally tailored video consists of unscripted narratives of black patients discussing their cancer diagnosis and CT experiences. The video spotlights Washington Cancer Institute (WCI) patients and their family members as well as members of the clergy, WCI physicians, ethicists, and staff, all of whom note the importance of black participation in CTs. Patients detail their experiences and any challenges related to each known barrier.

It is our belief that understanding the relationship between cultural attitudes and willingness to participate in CTs is key to improving accrual among black cancer patients. Many researchers have examined the lack of minority participation in CTs, but only a few have addressed the problem.^32, 34–41^ In a review of 50 studies conducted from 2001 to 2012 at an inner-city research center, it was found that effective recruitment strategies for black patients often involved a field-based approach; however, it was also noted that a majority of the research teams had members who understood the culture of the target population.^39^ The authors stated that cultural competence was critical in the design and implementation of successful recruitment strategies. While a culturally tailored video is an efficient tool to broach the topic of CTs and begin to dispel myths to change patient attitudes, more needs to be done to ensure ongoing education and awareness about the benefits of participating in clinical research, especially for minority patients.

Although it was not an endpoint we measured, it was clear that the patient navigation role the INSPIRE-BrC coordinators performed, from the time they enrolled the patients onto the study through the 6-month follow-up period, made an impact on patients’ attitudes and behaviors. Study coordinators checked in with patients by phone and in-person on a regular basis as part of study procedures, and as a way to build relationships. Study patients viewed INSPIRE-BrC coordinators as trusted resources, and relied on them to connect with other support services. Many patients who were invited to participate in a therapeutic trial not only consulted with their family members, friends and providers, but also with INSPIRE-BrC coordinators. Culturally and ethnically compatible research team members (e.g., patient navigators, study coordinators) have been shown to be an important aspect involved in improving minority CT participation.^32, 37, 39, 42^ In our study, one site, WCI, utilized consistent and racially congruent study coordinators, resulting in a 15.4% CT enrollment rate. Although other sites did not have strict consistency or racial congruency among study coordinators, the large majority of participants enrolled on INSPIRE-BrC came from WCI, likely diluting any negative impact on our study results (for site-specific enrollment refer to Supplementary Table 2).

Several potential study limitations are worthy of notation: (1) the non-randomized design that did not employ a control group; (2) the various stages of diagnosis and treatment at the time of participation may have impacted study participants’ attitudes toward therapeutic trials and intention/decision to enroll; (3) although men were not excluded from participating, our study represents only female patients’ perspectives; (4) a self-selection bias may exist, given the patients who agreed to participate in INSPIRE-BrC may have been likely to be less opposed to research in general; (5) participants were required to provide informed consent to take part in the study, which could have potentially influenced their responses to attitudinal measures on the AIET regarding consent; (6) there was no requirement that a trial was available at the time of participation, so at any given timepoint, a trial may not have been offered due to lack of trial availability for that given patient; and, (7) the overall sample size was small, providing limited power to detect nuanced differences in attitudes among this study population.

As denoted above, one limitation that may have hindered our findings was that therapeutic studies were only available for 27% of patients who participated in INSPIRE-BrC. It is likely that an even larger number of INSPIRE-BrC participants would have enrolled onto therapeutic trials had there been applicable studies available. This is not unusual as researchers have noted that study design issues (e.g., inclusion and exclusion criteria) and the lack of available trials open at any given institution at any given time contribute to the many barriers that hinder recruitment and accrual of patients, including minorities, to CTs.^7, 14, 43^ Based on this national issue, initiatives have been developed to streamline matching eligible patients to available trials nationwide.^44^

It is important to note that efforts were made to approach and enroll patients before they made treatment decisions. However, by nature of a within 6-month diagnosis eligibility requirement, some patients may have made treatment decisions prior to being approached and hence precluded their availability to participate in a trial after receiving the video intervention. We feel this pertains to only a few patients, however, as specific measures were made to present the study before any chemotherapy or radiation therapy decisions were made; and endocrine decisions at their primary diagnosis point if they had early stage disease. The INSPIRE-BrC research team worked extremely well with breast cancer tumor boards and other research coordinators within the MedStar system to ensure patients were prioritized. Additionally, the 6-month timeframe for eligibility was used to capture patients whose treatment was delayed or in the case of radiation trials, patients coming on trial after chemotherapy.

The accrual of racially and ethnically diverse populations to CTs remains one of the biggest challenges to advancing cancer care and improving clinical outcomes for these populations. Recognition of specific cultural barriers of these diverse populations and their relationship with CT participation is crucial to ameliorating disparities in accrual. Strategies, such as the use of a simple video intervention that can be widely disseminated and tailored to the specific culturally relevant barriers to recruitment and retention of minority populations into CTs, are critically needed. The results of this study are potentially promising for the utilization of a culturally targeted video to increase CT accrual among black cancer patients; however, despite an increase in willingness to participate in a CT, an actual increase in accrual was not observed and a controlled study of efficacy is warranted.

## Methods

### Intervention design

Eligible participants were identified through review of electronic clinical schedules, patient medical records, tumor boards or physicians, nurse navigators, clinical research coordinators and support services staff referrals at the following hospital sites: WCI at MedStar Washington Hospital Center; Lombardi Comprehensive Cancer Center at MedStar Georgetown University Hospital; MedStar Union Memorial Hospital; MedStar Harbor Hospital; and MedStar Franklin Square Medical Center (Supplementary Figure 1). Eligibility consisted of breast cancer patients who self-identified as black or African American (to include African, Caribbean or West Indian ancestry, or any other persons self-identifying as black), were aged 18 or older, and anticipated cancer treatment at one of the study locations. Patients must have been diagnosed within the past 6 months if stage I-III; or stage IV at any time. Only those able to understand and communicate verbally in English and give informed consent were invited to participate. Patients who had previously signed a consent form to participate in any type of therapeutic research study were ineligible. Any apparent physical distress or an altered mental status precluding the ability to give informed consent and/or complete study procedures was also basis for exclusion.

Participants were asked to commit at least an hour to complete initial study procedures on site. A study coordinator was paired with one participant at a time in a private setting. Prior to the intervention, a demographic survey was given to all participants to complete. The AIET questionnaire (Supplementary Table 1), a 31-item questionnaire developed to assess patient’s attitudes and intention to enroll in a therapeutic CT,^24^ was verbally administered by the study coordinator prior to watching the video (pre-test). The video was viewed in a private office setting via password protected computer access. The same questionnaire was administered again immediately following the video (post-test) and either in person or by phone between 7 and 21 days post video intervention (follow-up test). The patients’ verbal responses were recorded and entered into a secure electronic database; a program manager validated all data entries. Research coordinators followed patients for 6 months after the intervention to assess whether participants consented to and/or enrolled onto a therapeutic CT for which they were eligible. All aspects of the study were conducted in accordance with applicable international, national, and/or institutional guidelines and approved by the Georgetown/MedStar Health Institutional Review Board.

### Study measures and outcome variables

A pre-test/post-test method was utilized to determine the impact of the video on three variables—likely participation in therapeutic CTs; attitudes toward therapeutic CTs (assessed based on six barriers); and actual trial enrollment. The primary study outcome was the number of patients that participated in the video intervention who went on to sign consent/enroll on a therapeutic CT within 6 months.

Standard demographic data including age, marital status, and education level were collected. The AIET questionnaire was used to test intent to enroll and the six attitudinal barriers to participation: (1) Fear and distrust of the medical establishment/Trust in doctors; (2) Worry of being treated unfairly as a minority or poor patient (e.g. being a guinea pig); (3) Concern about ethical conduct of investigators; (4) Fear of losing one’s rights; (5) Loss of Privacy; (6) Lack of knowledge and awareness of CTs. Each of the attitudinal barriers represented a subscale on the AIET consisting of five items, each assessed on a 5-point Likert-type scale. Responses were scored as 1 = strongly disagree; 2 = somewhat disagree; 3 = not sure/neither; 4 = somewhat agree; 5 = strongly agree. The five-item responses for each attitudinal barrier were summed to produce a cumulative score with a possible range from 5 to 25. Some items were reverse-scored as appropriate. The last item on the questionnaire was used to assess a secondary outcome, patients’ hypothetical intention to enroll on a therapeutic CT: “At this moment, is it likely that you would sign up to participate in a therapeutic clinical trial?” The participants could only choose “Yes” or No” as a response and was the binary-dependent variable used for analysis.

### Statistical analyses

The primary outcome measure was the decision to participate in a therapeutic CT offered to the study participant after the intervention (Primary Objective). The percentage of participants who enrolled in a CT among all who were exposed to the intervention was compared to 6%, the 2012 MedStar baseline for black cancer patients; 22 out of the 384 black patients diagnosed with breast cancer that year enrolled in a therapeutic trial. A target sample size of 123 was initially chosen to provide 81% power to detect 6 percentage-point increase in participation rates (from a baseline rate of 6 to 12%) using a Type I error of 0.05 based on a one-sided binomial (EXACT) test. Based on a rapid accrual rate, it was decided to continue recruitment and enroll additional participants. At that time, the study was amended and a proposed sample size of 236 was selected to incorporate a 15% dropout rate to achieve a target of 200 patients. Based on this new sample size, an effect size of 4.8 could be detected.

Basic descriptive statistics were used to assess the distribution of socio-demographic and clinical characteristics in the study population. Bivariate associations between enrollment status and patient-level characteristics were examined by *t*-tests for continuous variables and chi-square and Fisher exact tests for categorical variables. McNemar and symmetry tests were used to examine changes in the responses to the attitudinal barriers and intention to enroll as a result of the video intervention. Given that every participant would be exposed to the intervention, logistic regression models were used to examine whether changes in attitudes (from pre-to-post-intervention and upon follow-up) significantly influenced the actual decision to participate in a CT. Multiple imputations were used when necessary for missing data. Two-tailed *p* < .05 was considered statistically significant. Statistical analyses were performed using SAS, 9.4 (SAS Institute, Cary, NC).

### Data availability

Available upon request. Please contact the corresponding author.

### Link to Video Intervention

“Today’s Truth: Research Brings Hope - INcreaSing Participation In Research-Breast Cancer” by MedStar Health Research Institute is available online at https://www.MedStarResearch.org/INSPIRE and is licensed under a Creative Commons Attribution 4.0 International License.

## Electronic supplementary material

Supplementary Table 1. Attitudes and Intention to Enroll in Therapeutic Clinical Trials (AIET) Survey

Supplementary Table 2. INSPIRE-BrC Clinical Trial Enrollment by Site

Supplementary Figure 1. Flow Chart of Study Activities Relating to Patient Recruitment
