# Supplementary material for: Video intervention increases participation of black breast cancer patients in therapeutic trials
Source: NPJ Breast Cancer. 2017 Sep 18;3:36. doi: 10.1038/s41523-017-0039-1 (PMC5603544; doi:10.1038/s41523-017-0039-1)
Supplement: Supplementary file 1 — Supplementary Table 1. Attitudes and Intention to Enroll in Therapeutic Clinical Trials (AIET) Survey [file 41523_2017_39_MOESM1_ESM.docx]

**Supplementary Online Content**

**Video Intervention Increases Participation of Black Breast Cancer Patients in Therapeutic Trials**

**Table 1.** The Attitudes and Intention to Enroll in Therapeutic Clinical Trials (AIET) Survey

**Table 1.** Attitudes and Intention to Enroll in Therapeutic Clinical Trials (AIET) Survey

**MHRI GU IRB # 2014-0018**

**Title:** IncreaSing Participation in Research – Breast Cancer (INSPIRE-BrC)

**Attitudes and Intention to Enroll in Therapeutic Clinical Trials Survey**

MR#: ____________________ Study ID#_____________________

Name: ___________________________

For each statement below, please circle the number between 1 and 5 that represents how you feel about each statement:

| 1. | Trust in the doctor who offers you the trial is important  1=Not at all 2=A little 3=Not Sure/Neither 4=Some 5=Very much |
| --- | --- |
| 2. | The reputation of the treatment center where the trial is done is important  1=Not at all 2=A little 3=Not Sure/Neither 4=Some 5=Very much |
| 3. | I cannot trust health care workers  1=Strongly Disagree 2= Somewhat Disagree 3=Not Sure/Neither 4=Somewhat Agree 5=Strongly Agree |
| 4. | I am suspicious of clinical trials  1=Strongly Disagree 2= Somewhat Disagree 3=Not Sure/Neither 4=Somewhat Agree 5=Strongly Agree |
| 5. | I am suspicious of information I receive from researchers  1=Strongly Disagree 2= Somewhat Disagree 3=Not Sure/Neither 4=Somewhat Agree 5=Strongly Agree |
| 6. | Most clinical research is ethical  1=Strongly Agree 2=Somewhat Agree 3=Not sure/Neither 4=Somewhat Disagree 5=Strongly Disagree |
| 7. | Researchers do not care about me or my well being  1=Strongly Disagree 2= Somewhat Disagree 3=Not Sure/Neither 4=Somewhat Agree 5=Strongly Agree |
| 8. | My doctor would not ask me to participate in a clinical trial if he or she thought it would hurt me  1=Strongly Agree 2=Somewhat Agree 3=Not sure/Neither 4=Somewhat Disagree 5=Strongly Disagree |
| 9. | I am confident the group of people who approve clinical trials make sure all participants are treated fairly  1=Strongly Agree 2=Somewhat Agree 3=Not sure/Neither 4=Somewhat Disagree 5=Strongly Disagree |
| 10. | How likely do you think it is that you might be used as a guinea pig if you were in a clinical trial?  1=Not at all likely 2=Somewhat unlikely 3=Not sure/Neither 4=Somewhat likely 5=Very likely |
| 11. | I could still ask my doctors any questions that I want to  1=Strongly Agree 2=Somewhat Agree 3=Not sure/Neither 4=Somewhat Disagree 5=Strongly Disagree |
| 12. | If doctors took my blood they could do tests on it they have not told me about  1=Strongly Disagree 2= Somewhat Disagree 3=Not Sure/Neither 4=Somewhat Agree 5=Strongly Agree |
| 13. | I would only be agreeing to do what is explained to me in the consent form  1=Strongly Agree 2=Somewhat Agree 3=Not sure/Neither 4=Somewhat Disagree 5=Strongly Disagree |
| 14. | I could still change my mind about participating at any time  1=Strongly Agree 2=Somewhat Agree 3=Not sure/Neither 4=Somewhat Disagree 5=Strongly Disagree |
| 15. | The researchers would only do what is stated in the consent form  1=Strongly Agree 2=Somewhat Agree 3=Not sure/Neither 4=Somewhat Disagree 5=Strongly Disagree |
| 16. | Black people in clinical trials receive the same care from doctors and health care workers as people of other races or ethnicities on clinical trials  1=Strongly Agree 2=Somewhat Agree 3=Not sure/Neither 4=Somewhat Disagree 5=Strongly Disagree |
| 17. | If I were to enroll in a clinical trial my doctors would treat me with dignity and respect  1=Strongly Agree 2=Somewhat Agree 3=Not sure/Neither 4=Somewhat Disagree 5=Strongly Disagree |
| 18. | Compared with others, poor people are used more in research without their permission  1=Strongly Disagree 2= Somewhat Disagree 3=Not Sure/Neither 4=Somewhat Agree 5=Strongly Agree |
| 19. | How often, if ever, do you think doctors prescribe medication as a way of experimenting on Black patients without their knowledge or permission?  1=Never 2=Rarely 3=Sometimes 4=Often 5=Very often |
| 20. | Black people are used more in research without their knowledge or permission than others races and ethnicities  1=Strongly Disagree 2= Somewhat Disagree 3=Not Sure/Neither 4=Somewhat Agree 5=Strongly Agree |
| 21. | People can access my medical records without my approval.  1=Strongly Disagree 2= Somewhat Disagree 3=Not Sure/Neither 4=Somewhat Agree 5=Strongly Agree |
| 22. | My medical records are kept private.  1=Strongly Agree 2=Somewhat Agree 3=Not sure/Neither 4=Somewhat Disagree 5=Strongly Disagree |
| 23. | My privacy is a major concern for the researchers involved  1=Strongly Agree 2=Somewhat Agree 3=Not sure/Neither 4=Somewhat Disagree 5=Strongly Disagree |
| 24. | Personal information like my name, address and phone number will remain confidential  1=Strongly Agree 2=Somewhat Agree 3=Not sure/Neither 4=Somewhat Disagree 5=Strongly Disagree |
| 25. | Any center doing clinical trials has set rules to make sure my records are kept safe  1=Strongly Agree 2=Somewhat Agree 3=Not sure/Neither 4=Somewhat Disagree 5=Strongly Disagree |
| 26. | There are always serious side effects related to clinical trials  1=Strongly Disagree 2= Somewhat Disagree 3=Not Sure/Neither 4=Somewhat Agree 5=Strongly Agree |
| 27. | If my doctor wanted me to participate in a clinical trial, he or she would fully explain to me everything that is involved  1=Strongly Agree 2=Somewhat Agree 3=Not sure/Neither 4=Somewhat Disagree 5=Strongly Disagree |
| 28. | I can talk to my doctors to find out about participating in clinical trials  1=Strongly Agree 2=Somewhat Agree 3=Not sure/Neither 4=Somewhat Disagree 5=Strongly Disagree |
| 29. | There may be benefits for me if I participate in a clinical trial  1=Strongly Agree 2=Somewhat Agree 3=Not sure/Neither 4=Somewhat Disagree 5=Strongly Disagree |
| 30. | There may be benefits for other people like me if I participate in a clinical trial  1=Strongly Agree 2=Somewhat Agree 3=Not sure/Neither 4=Somewhat Disagree 5=Strongly Disagree |
| *31a.* | *At this moment, is it likely that you would sign up to participate in a therapeutic clinical trial?*  *Yes No* |
| *31b.* | *(Same Question) At this moment, is it likely that you would sign up to participate in a therapeutic clinical trial?*  *Yes No* |
| *31c.* | *(Same Question) At this moment, is it likely that you would sign up to participate in a therapeutic clinical trial?*  *Yes No* |
