# Supplementary material for: Video intervention increases participation of black breast cancer patients in therapeutic trials
Source: NPJ Breast Cancer. 2017 Sep 18;3:36. doi: 10.1038/s41523-017-0039-1 (PMC5603544; doi:10.1038/s41523-017-0039-1)
Supplement: Supplementary file 2 — Supplementary Table 2. INSPIRE-BrC Clinical Trial Enrollment by Site [file 41523_2017_39_MOESM2_ESM.docx]

**Supplementary Online Content**

**Video Intervention Increases Participation of Black Breast Cancer Patients in Therapeutic Trials**

**Table 2.** INSPIRE-BrC Clinical Trial Enrollment by Site

**Table 2.** INSPIRE-BrC Clinical Trial Enrollment by Site

| MedStar Hospital | Patients enrolled on INSPIRE-BrC | INSPIRE-BrC participants enrolled on therapeutic studies | Percentage enrolled on trials by site |
| --- | --- | --- | --- |
| MedStar Franklin Square Medical Center | 11 | 0 | 0.0 |
| MedStar Georgetown University Hospital | 22 | 3 | 13.6 |
| MedStar Harbor Hospital | 13 | 0 | 0.0 |
| MedStar Union Memorial Hospital | 18 | 3 | 16.7 |
| MedStar Washington Hospital Center | 136 | 21 | 15.4 |
| Total | **200** | **27** | **13.5** |

Abbreviations: INSPIRE-BrC, INcreaSing Participation In Research-Breast Cancer
