# Supplementary material for: Video intervention increases participation of black breast cancer patients in therapeutic trials
Source: NPJ Breast Cancer. 2017 Sep 18;3:36. doi: 10.1038/s41523-017-0039-1 (PMC5603544; doi:10.1038/s41523-017-0039-1)
Supplement: Supplementary file 3 — Supplementary Figure 1. Flow Chart of Study Activities Relating to Patient Recruitment [file 41523_2017_39_MOESM3_ESM.docx]

**Supplementary Online Content**

**Video Intervention Increases Participation of Black Breast Cancer Patients in Therapeutic Trials**

**Figure 1.** Flow Chart of Study Activities Relating to Patient Recruitment

**Figure 1.** Flow Chart of Study Activities Relating to Patient Recruitment

**
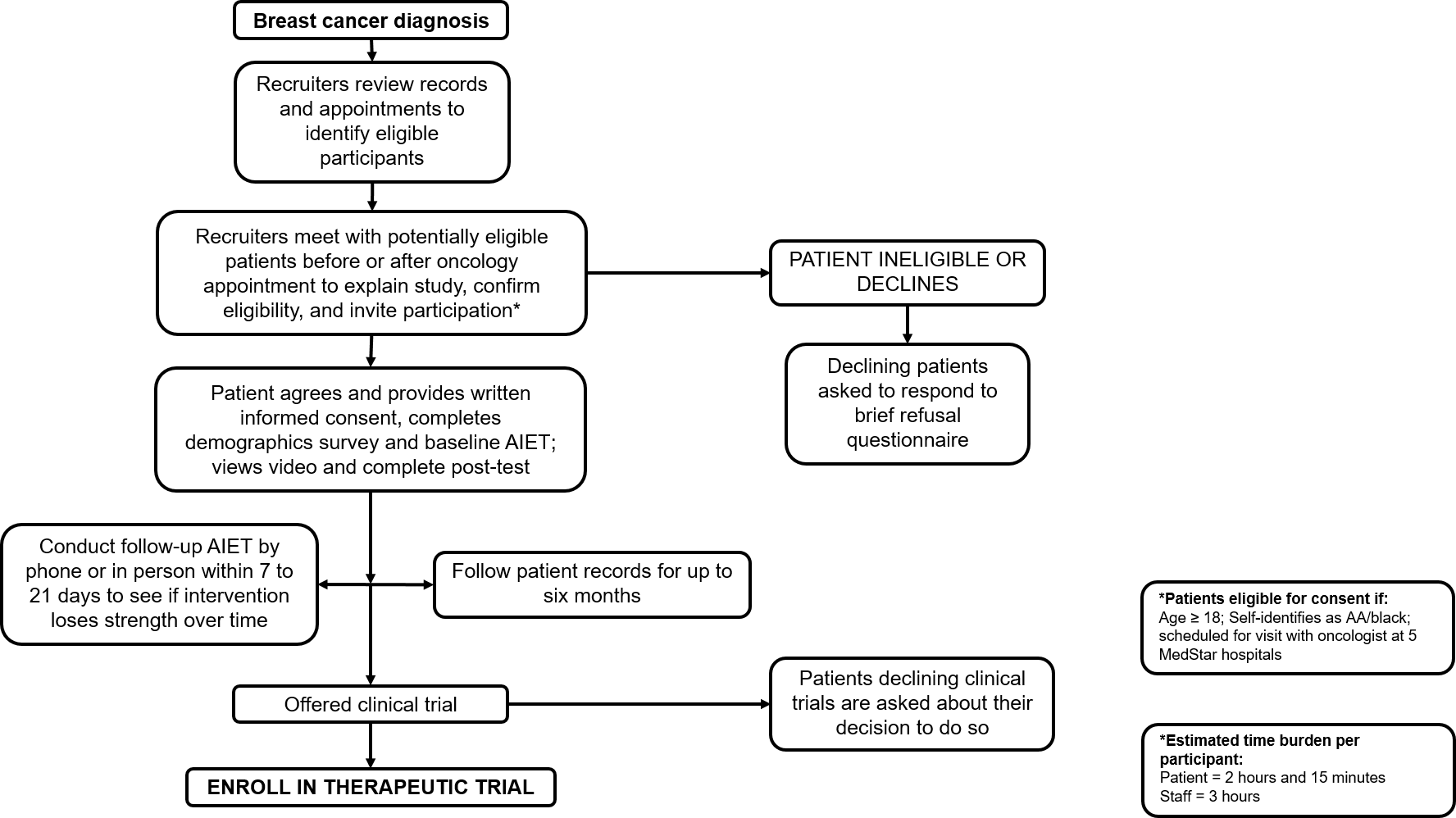
**

Abbreviations: AA, African American; AIET, Attitudes and Intention to Enroll in Therapeutic Clinical Trials questionnaire.
